# Gene network approach reveals co-expression patterns in nasal and bronchial epithelium

**DOI:** 10.1038/s41598-019-50963-x

**Published:** 2019-11-01

**Authors:** Kai Imkamp, Victor Bernal, Marco Grzegorzcyk, Peter Horvatovich, Cornelis J. Vermeulen, Irene H. Heijink, Victor Guryev, Huib A. M. Kerstjens, Maarten van den Berge, Alen Faiz

**Affiliations:** 1 University of Groningen, University Medical Center Groningen, Department of Pulmonology, Groningen, The Netherlands; 2 University of Groningen, University Medical Center Groningen, GRIAC (Groningen Research Institute for Asthma and COPD), Groningen, The Netherlands; 3 University of Groningen, Bernoulli Institute (JBI), Groningen, The Netherlands; 4 University of Groningen, Department of Pharmacy, Analytical Biochemistry, Groningen, The Netherlands; 5 University of Groningen, University Medical Center Groningen, Department of Pathology & Medical Biology, section Medical Biology, Groningen, The Netherlands; 6 European Research Institute for the Biology of Ageing, University of Groningen, University Medical Center Groningen, Groningen, The Netherlands; 7 University of Technology Sydney, Respiratory Bioinformatics and Molecular Biology (RBMB), School of life sciences, Sydney, Australia; 8 Woolcock Emphysema Centre, Woolcock Institute of Medical Research, University of Sydney, Sydney, NSW Australia

**Keywords:** Chronic obstructive pulmonary disease, Genetics research

## Abstract

Nasal gene expression profiling is a new approach to investigate the airway epithelium as a biomarker to study the activity and treatment responses of obstructive pulmonary diseases. We investigated to what extent gene expression profiling of nasal brushings is similar to that of bronchial brushings. We performed genome wide gene expression profiling on matched nasal and bronchial epithelial brushes from 77 respiratory healthy individuals. To investigate differences and similarities among regulatory modules, network analysis was performed on correlated, differentially expressed and smoking-related genes using Gaussian Graphical Models. Between nasal and bronchial brushes, 619 genes were correlated and 1692 genes were differentially expressed (false discovery rate <0.05, |Fold-change|>2). Network analysis of correlated genes showed pro-inflammatory pathways to be similar between the two locations. Focusing on smoking-related genes, cytochrome-P450 pathway related genes were found to be similar, supporting the concept of a detoxifying response to tobacco exposure throughout the airways. In contrast, cilia-related pathways were decreased in nasal compared to bronchial brushes when focusing on differentially expressed genes. Collectively, while there are substantial differences in gene expression between nasal and bronchial brushes, we also found similarities, especially in the response to the external factors such as smoking.

## Introduction

Chronic obstructive pulmonary disease (COPD) is a chronic inflammatory obstructive disorder of the airways that affects millions of people worldwide^1^. It is a complex and heterogeneous disease caused by many factors, including environmental particles and genetic factors leading to inflammation and metabolic disturbances. Currently COPD is the third most lethal disease worldwide according to World Health Organization^1^. It is mainly caused by inhalation of noxious particles e.g. cigarette smoking, air pollution or indoor cooking, but the disease onset and severity depend on genetic predisposition of the person affected by these environmental circumstances^2–4^.

The initial site of exposure to inhaled substances and particles is the airway epithelium and our group and others have demonstrated that airway gene expression signatures can serve as biomarkers to assess the activity/severity of COPD and asthma^5,6^. It has also been shown that chronic exposure to tobacco smoke results in both reversible and irreversible changes in bronchial airway epithelial gene expression, a so-called ‘airway field of injury’^7,8^. Furthermore, we identified a bronchial gene expression signature that is associated with COPD alteration and disease severity with similar gene expression changes in lung tissue affected by COPD^5^. These data allowed the first finding, which implied that bronchial gene expression obtained by bronchoscopy could be used to assess the disease activity in COPD. However, bronchoscopy is an invasive procedure with a substantial burden to the patient, preventing its use in large populations as well as frequent sampling.

We previously demonstrated that the nasal epithelium can be used to detect changes in COPD-associated gene expression and showed that the nasal epithelial COPD related gene expression signature partly overlaps with COPD-associated bronchial epithelial gene expression^9^. This strengthens the hypothesis that the upper and lower airways have a common COPD-related gene expression profile, the united ‘airway field of injury’. Previously, we and others have shown that nasal and bronchial epithelium have a similar expression profile in smokers and never-smokers. Additionally, we have shown that expression quantitative trait loci (eQTL) are similar between the two compartments^10^. The easy availability to nasal epithelium, nasal gene expression provides the opportunity to assess disease-related phenotypes and determine treatment outcome.

One way to investigate this is the use of gene network modelling. In particular, Gaussian Graphical Models (GGMs), are a widely used network model to study protein^11^, and gene regulatory networks^12^. The GGM consists of nodes (e.g. genes, proteins or metabolites) interconnected by edges if their partial correlation is significantly different from zero. Partial correlations are correlations where the cofounding effects are removed. This is an advantage of GGMs compared to other models such as Relevance Networks^13^ or weighted gene co-expression network analysis (WGCNA)^14^, principal component analysis or clustering, which use the structure obtained from Pearson correlation to find similarity patterns because spurious associations are avoided. For example, a common regulator gene might results in similar expression patterns of the regulated genes. These patterns are then indirect and cannot be distinguished from the effect of the regulator. Learning the structure of a GGM (i.e. inferring the edges from data) is computationally feasible even for large networks, and often performs as good as other more demanding network models (e.g. Bayesian Networks)^15^. Some applications in respiratory research include GGMs reconstructed from expression data of asthmatic children^16^, asthma integrated genomic data^17^, COPD phenotypic networks^18^, and asthma gene – single-nucleotide polymorphism (SNP) associations^19^.

In the current study, we aim to investigate to what extent gene expression profiling of nasal brushings are similar to that of bronchial brushings, and to determine whether nasal brushing can be used as a non-invasive biomarker of the lower airways in the study of respiratory diseases. We used GGMs to investigate correlated and differentially expressed genes between the two tissues, and to identify which pathways are similar and which are different.

## Results

### Patient characteristics

From the 110 respiratory healthy participants who were enrolled in the study, 77 had matched nasal and bronchial samples. Table 1 shows the clinical characteristics of all participants and the comparison of current and never smokers.

**Table 1:** Clinical characteristics of the NORM study.

	All (N = 77)	Current smokers (N = 41)	Never smokers (N = 36)
Age, yr	36.06 (16.23)	37.10 (15.55)	34.89 (17.10)
BMI kg/m^2^	23.73 (3.50)	23.91 (3.29)	23.52 (3.76)
Gender, Male/Female	41/36	25/16	16/20
Pack years****	8.68 (13.4)	16.30 (14.63)	0
FEV_1_% predicted	108.14 (10.49)	106.73 (10.62)	109.75 (10.24)
Reversibility % from baseline	3.82 (3.05)	3.82 (2.64)	3.82 (3.50)
FEV_1_/FVC	83.06 (6.37)	81.75 (5.97)	84.54 (6.57)
RV % predicted	93.74 (17.46)	92.83 (12.99)	94.78 (21.62)
TLC % predicted	104.04 (9.42)	102.80 (9.56)	105.44 (9.19)
RV/TLC % predicted	85.62 (12.38)	85.95 (8.84)	85.25 (15.59)

BMI, body mass index; FEV_1_, forced expiratory volume in one second; FEV_1_/FVC, forced expiratory volume in one second/forced vital capacity; RV, residual volume; TLC, total lung capacity; RV/TLC, residual volume/total lung capacity.

The means and standard deviations are shown for continuous variables. ****Independent T-test showed significant difference between the two groups only with a p < 0.0001.

### Genes similar between the nose and the bronchus

**Figure 1:**
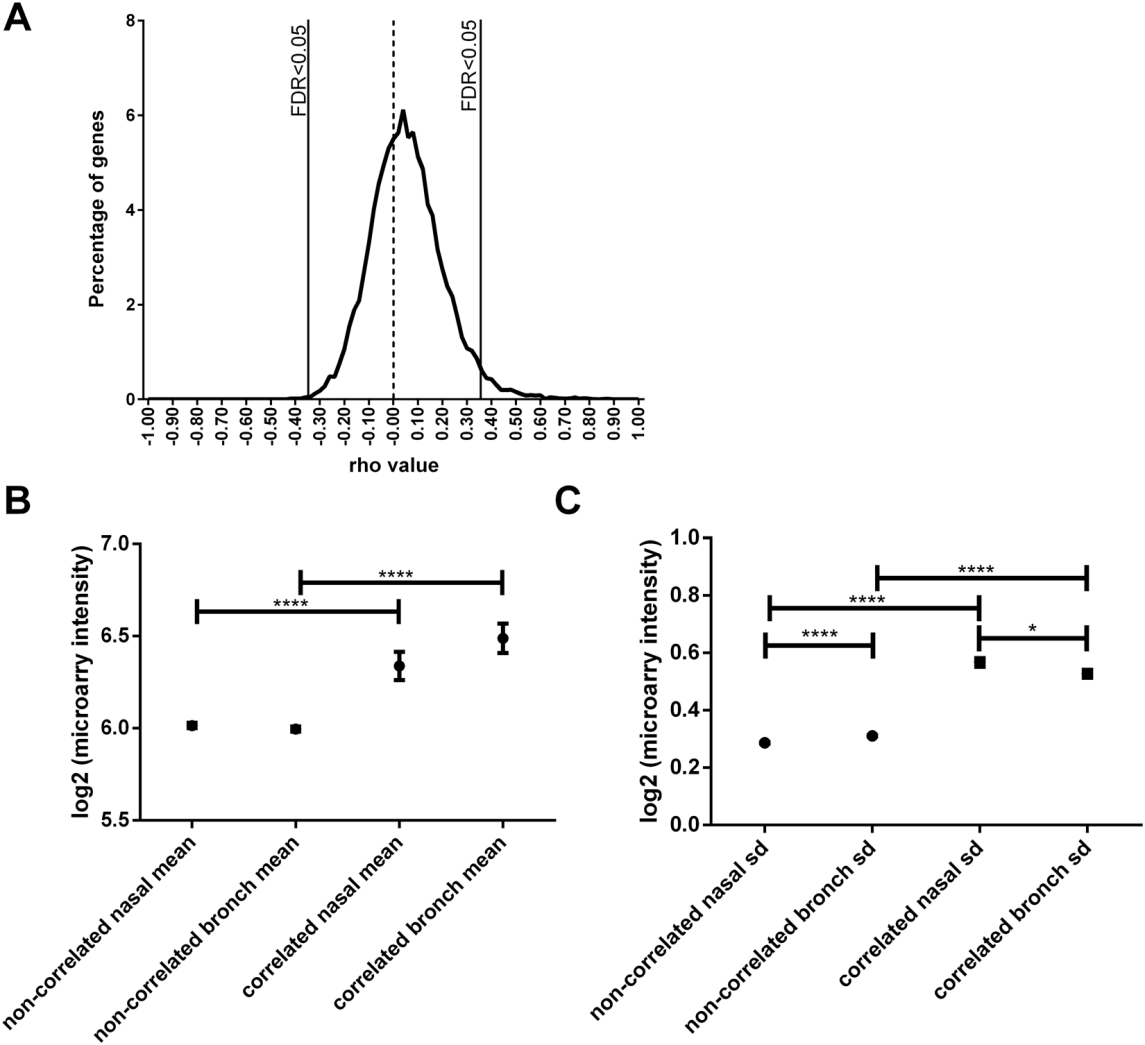
Comparison between nasal and bronchial epithelial brushes. (**A**) Histogram of Spearman correlation of each concordant gene (FDR-adjusted p < 0.05) between nasal and bronchial brushes. (**B**) Comparing mean expression from correlated and non-correlated genes (**C**) Comparing standard error. SD = standard error.

**Figure 2:**
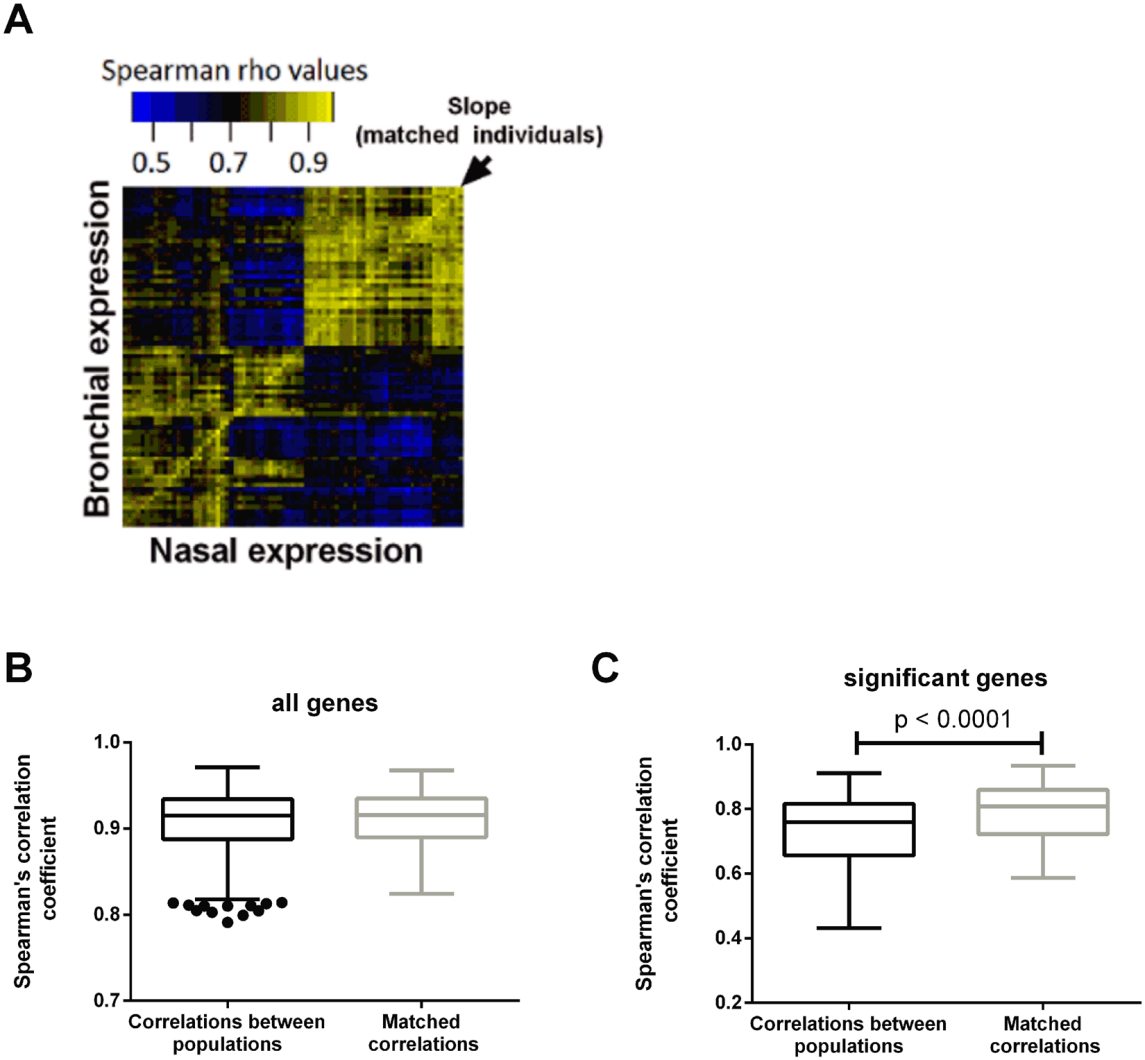
Correlation between nasal and bronchial epithelial brushes. (**A**) Heatmap showing Spearman correlation of genes between nasal and bronchial brushes (FDR-adjusted p < 0.05). (**B**) Correlation between independent and matched samples using all genes and (**C**) genes which correlate between matched samples of nasal and bronchial brushes.

Spearman correlation analyses, comparing the expression of individual genes between nasal and bronchial brushes, identified 619 genes (3.4% of total, Benjamini-Hochberg (BH)^20^ -adjusted p < 0.05) that were significantly correlated between bronchial and nasal samples. Table 2 shows the top 20 genes with correlated expression between the nasal and the bronchial epithelium. As expected, X- and Y-linked genes, smoking-related genes, and genes known to have high genetic contribution to variation in expression, such as *HLA-DRB1*, were the most significantly correlated genes in this analysis^21^. For the majority of these genes (98%) expression was positively correlated between nasal and bronchial brushes (Fig. 1A), indicating a subset of genes with strong concordance of their expression between the two sampling locations. To confirm that these findings were not by chance, a permutation analysis was conducted (n = 500). Indeed, the number of positively correlated genes was always found to be greater with paired samples compared to randomly picked (non-paired) samples (p < 0.002). Furthermore, we found that genes correlated between the two locations have both higher mean expression and greater variation (standard deviation) than non-correlated genes (Fig. 1B,C), indicating that variation of gene expression is required for correlation. We next investigated to what extent the nasal sample reflects an individual’s bronchial transcriptional profile rather than a response to environmental insults such as smoking. To this end, we assessed whether the nasal-bronchial relation within a patient was stronger than across patients. We performed this analysis for all genes genome-wide and for the list of 619 correlated genes mentioned above. We found no difference with respect to the nasal-bronchial relation between samples from the nose and bronchus when looking at all genes, while for the 619 correlated genes (CO), the intra-patient nasal bronchial correlations were more correlated than across patients (Fig. 2A–C). This may be explained by the low expression or low variation in expression in the population of the non-correlating genes. These two factors drive the lack of self-correlation as the levels of expression between nasal and bronchial brushes are very similar across all patients.

**Table 2:** Top 20 genes correlated between nasal and bronchial brushes (FDR < 0.05).

Gene symbol	rho	p-value^**^	FDR^**^
PSORS1C3	0.649	0	0
TEKT4P2	0.658	0	0
CYP1A1*	0.652	0	0
CYP1B1*	0.774	0	0
WBSCR27	0.653	0	0
CFD	0.673	0	0
SLC44A5	0.690	0	0
NLGN4Y	0.730	0	0
SLC7A11*	0.648	0	0
TXLNG2P	0.720	0	0
TAS2R43	0.787	0	0
GBP3	0.880	0	0
RNU5D-1	0.641	0	0
STEAP1	0.727	0	0
ABO	0.745	0	0
GSTM1	0.743	0	0
GSTT1	0.798	0	0
GUCY1B2	0.667	0	0
HLA-DMB	0.633	0	0
HLA-DQA1	0.787	0	0

*Smoking related genes.

**Zero (0) p-values and FDR rate means that the number is smaller than the smallest number that can be represented by a double format in R (i.e. <10^−308^).

### Network analysis on correlated genes

**Figure 3:**
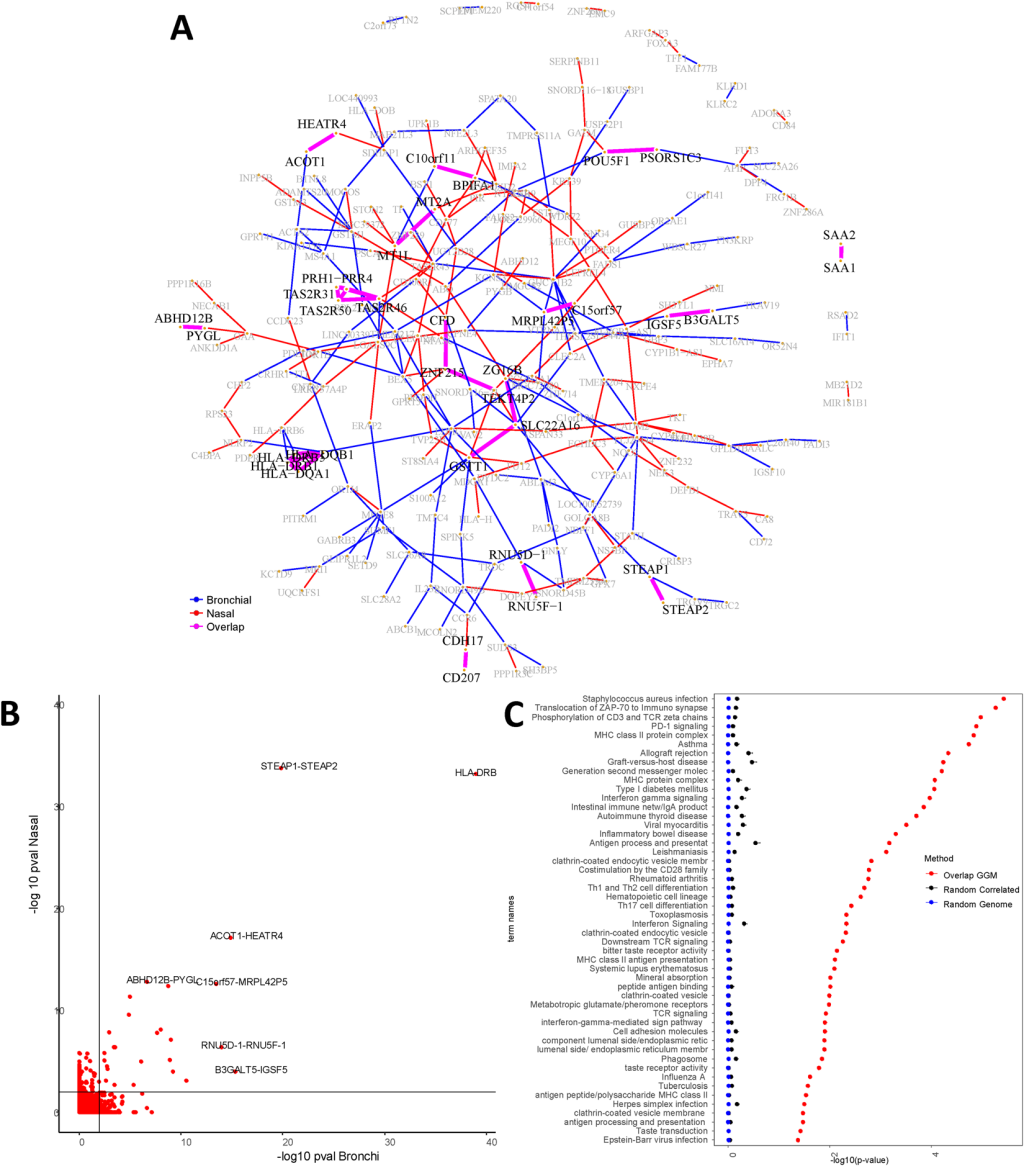
Network analyses for the correlated (CO) genes. (**A**) GGM network for the CO genes (BH $p\le 0.01$). Genes that had no significant connections (partial correlation) were left out of the figure, and the genes connected in only one of the tissues are colored in grey. In blue: edges present in bronchial tissue. In red: edges present in nasal tissue. In magenta: edges present both tissues (*Overlapped* edges). (**B**) Scatter plot of the GGM network edges (partial correlation) for the CO genes. Each red dot represents $-lo{g}_{10}(\text{BH}\,p)$ of an edge in bronchial tissue (horizontal axis) versus nasal tissue (vertical axis). In light black: the critical value at BH $p\le \mathrm{0.01.}$ The figure displays the respective gene pairs for the most similar edges. (**C**) The 50 most significant GOs for the set of *Overlapped* genes. The enrichment is contrasted against two sets of genes randomly sampled from the CO (619 genes) and from the whole genome (19718 genes). The panel displays the corresponding mean $-lo{g}_{10}$ (p-values) and error bars represent + 2 standard errors over the 500 random samples.

From these 619 CO genes, we built two GGM networks (one for each tissue) at BH-adjusted *p* ≤ 0.01 (Fig. 3A). We found 163 genes (26.33%) that are connected in the bronchial network with 156 edges (i.e. significant partial correlations), and 168 (27.14%) in nasal network with 152 edges. In total 236 genes (38.12%) had at least one edge in one of the tissues, from which only 36 genes (15.25%) have common edges in both compartments.

Figure 3B shows an (edge-wise) comparison of the networks via a scatter plot of BH p-values for the CO genes. We observed that genes belonging to the same family tend to be interconnected in both tissues. In particular, *HLA-DQB1*, *HLA-DRB1*, *HLA-DRB5* and *HLA-DQA1* were interconnected to each other. Other gene pairs from the same family are connected as well, namely; (*i*) *SAA1-SAA*2, (*ii) STEAP1-STEAP2*, (*iii) RNU5F-1 - RNU5D-1*, (*iv) MT2A - MT1L*, (*v) CD207- CDH17*. Table 3 shows the results of GO enrichment analysis for biological processes at False Discovery Rate (FDR) ≤0.05 (Fig. 3C). The enrichment consisted of 56 biological processes mainly related to inflammatory and immunological pathways.

**Table 3:** List of top 10 enriched GOs for the overlapping genes set from the CO genes ($FDR\le 0.05$).

Term ID	Term name	p-value	Intersection
KEGG:05150	Staphylococcus aureus infection	3.63·10^−6^	HLA-DQB1, HLA-DRB1, HLA-DQA1, CFD, HLA-DRB5
REAC:202430	Translocation of ZAP-70 to Immunological synapse	5.28·10^−6^	HLA-DQB1, HLA-DRB1, HLA-DQA1, HLA-DRB5
REAC:202427	Phosphorylation of CD3 and TCR zeta chains	1.03·10^−5^	HLA-DQB1, HLA-DRB1, HLA-DQA1, HLA-DRB5
REAC:389948	PD-1 signaling	1.26·10^−5^	HLA-DQB1, HLA-DRB1, HLA-DQA1, HLA-DRB5
GO:0042613	MHC class II protein complex	1.43·10^−5^	HLA-DQB1, HLA-DRB1, HLA-DQA1, HLA-DRB5
KEGG:05310	Asthma	1.77·10^−5^	HLA-DQB1, HLA-DRB1, HLA-DQA1, HLA-DRB5
KEGG:05330	Allograft rejection	4.48·10^−5^	HLA-DQB1, HLA-DRB1, HLA-DQA1, HLA-DRB5
KEGG:05332	Graft-versus-host disease	5.63·10^−5^	HLA-DQB1, HLA-DRB1, HLA-DQA1, HLA-DRB5
REAC:202433	Generation of second messenger molecules	6.11·10^−5^	HLA-DQB1, HLA-DRB1, HLA-DQA1, HLA-DRB5
GO:0042611	MHC protein complex	8.27·10^−5^	HLA-DQB1, HLA-DRB1, HLA-DQA1, HLA-DRB5

### Network analysis on smoking-related genes

**Figure 4:**
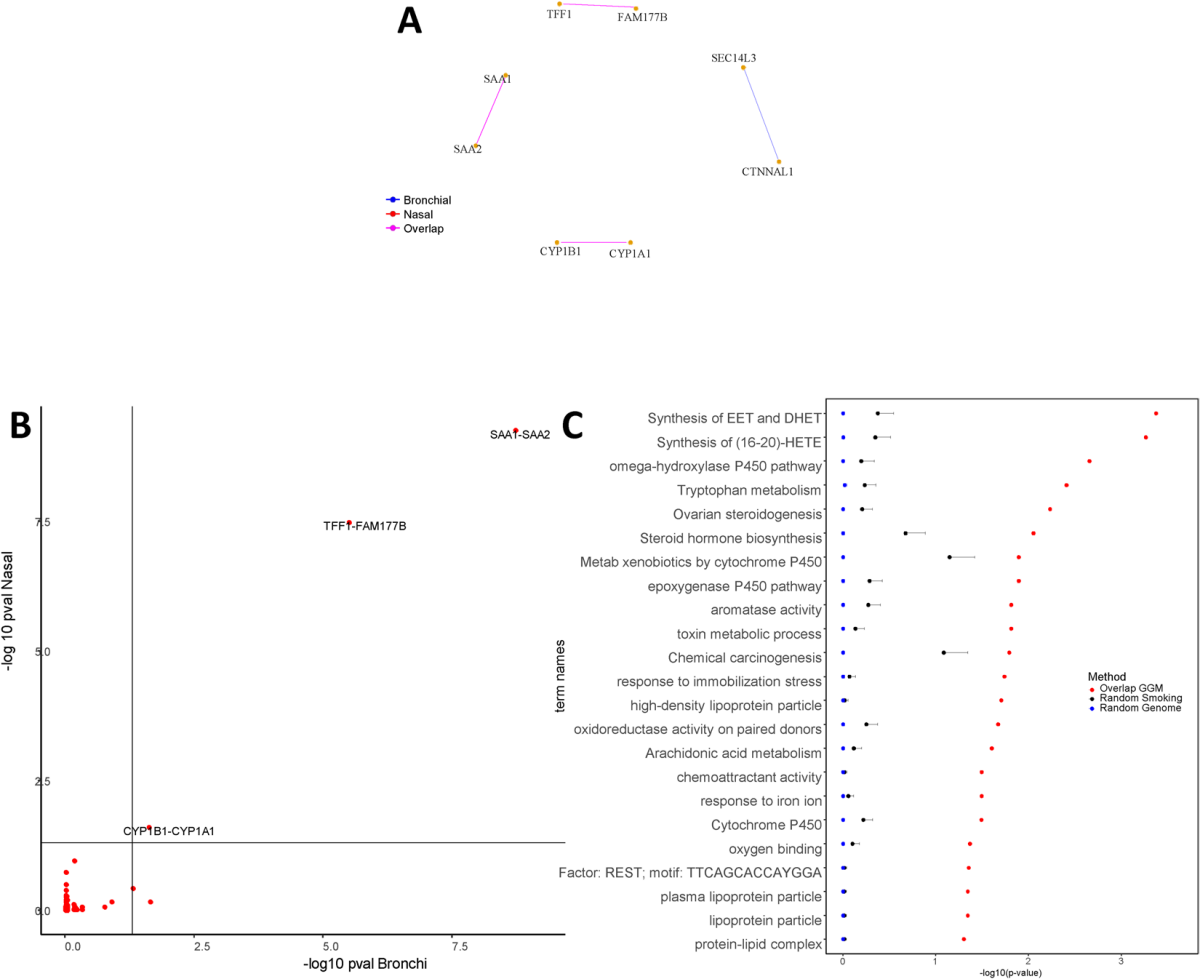
Network analyses for the smoking (SM) genes. (**A**) GGM network for the SM genes (BH $p\le 0.05\,$). Genes which had no significant connections (partial correlation) were left out of the figure, and the genes connected in only one of the tissues are colored in grey. In blue: edges present in bronchial tissue. In red: edges present in nasal tissue. In magenta: edges present both tissues (*Overlapped* edges). (**B**) Scatter plot of the GGM network edges (partial correlation) for the SM genes. Each red dot represents $-lo{g}_{10}(\text{BH}\,p)$ of an edge in bronchial tissue (horizontal axis) versus nasal tissue (vertical axis). In light black: the critical value at BH $p\le 0.05\,.$ The figure displays the respective gene pairs for the most similar edges. (**C**) 22 significant GOs for the set of *Overlapped* genes. The enrichment is contrasted against two sets of genes randomly sampled from the SM (27 genes) and from the whole genome (19718 genes). The panel displays the corresponding mean $-lo{g}_{10}$ (p-values), and error bars represent + 2 standard errors over the 500 random samples.

Previously, we identified 27 genes that were differentially expressed in current smokers compared to never smokers in both the nasal and bronchial brushes^22^. From these 27 smoking-related genes (SM), we inferred two GGM networks (one for each tissue) at BH-adjusted ≤0.05 (Fig. 4A). We found 8 genes (29.62%) that are connected in the bronchial network with 4 edges (i.e. having multiple significant partial correlations), and 6 genes (22.22%) in nasal network with 3 edges. A total of 6 genes (75.00%, 3 gene pairs) exhibited one common edge between the bronchial and nasal network. Figure 4B shows an (edge-wise) comparison of the networks via a scatter plot of BH p-values for the SM genes. We observed that two gene pairs belonging to the same family are connected in both tissues, namely; *(i) SAA1-SAA2*, *(ii) CYP1A1-CYP1B1* and *(iii) TFF1- FAM177B*. The enrichment was observed for 22 biological processes mainly related to the metabolism of xenobiotics and other P450 related pathways (Fig. 4C). Table 4 shows the results of GO enrichment analysis for biological processes at FDR ≤ 0.05.

**Table 4:** List of top 10 enriched GOs for the overlapping genes set from the SM genes ($FDR\le 0.05$).

Term ID	Term name	p-value	Intersection
REAC:2142670	Synthesis of epoxy (EET) and dihydroxyeicosatrienoic acids (DHET)	0.000211	CYP1B1, CYP1A1
REAC:2142816	Synthesis of (16–20)-hydroxyeicosatetraenoic acids (HETE)	0.000271	CYP1B1, CYP1A1
KEGG:00380	Tryptophan metabolism	0.000539	CYP1B1, CYP1A1
KEGG:04913	Ovarian steroidogenesis	0.000847	CYP1B1, CYP1A1
KEGG:00140	Steroid hormone biosynthesis	0.00114	CYP1B1, CYP1A1
KEGG:00980	Metabolism of xenobiotics by cytochrome P450	0.00177	CYP1B1, CYP1A1
GO:0097267	omega-hydroxylase P450 pathway	0.00214	CYP1B1, CYP1A1
KEGG:05204	Chemical carcinogenesis	0.00224	CYP1B1, CYP1A1
GO:0009404	toxin metabolic process	0.00623	CYP1B1, CYP1A1
GO:0019373	epoxygenase P450 pathway	0.0125	CYP1B1, CYP1A1

### Transcription profiles differ between the nose and the bronchus

**Figure 5:**
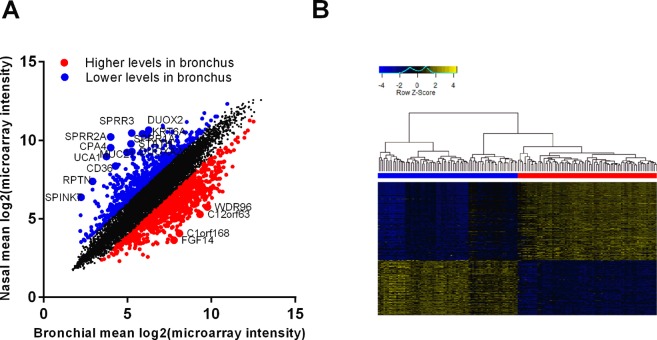
Differences between nasal and bronchial epithelial brushes. (**A**) Correlation plot of the mean gene expression from nasal and bronchial brushes with genes significantly lower and higher in bronchial brushes highlighted in blue and red, respectively (FDR < 0.05). (**B**) Heatmap of gene differentially expressed between nasal (blue) and bronchial (red) brushes (|Log_2_FC| > 1.5, FDR < 0.05).

Next, we investigated which genes drive the differences between the locations. We identified 6,806 expressed in nasal and 6,797 genes expressed in bronchial epithelium (log_2_(microarray florescence) <3), with an overlap of 98.2%. Expression of 130 genes was specific to bronchial epithelium, while 145 genes were specifically expressed in nasal epithelium. A differential expression analysis identified 1692 differential expressed genes (DEGs), among which 723 (3.98%) DEGs with higher and 969 (5.34%) with lower expression (|log fold change|>2, FDR < 0.05) in nasal compared to bronchial brushes. Table 5 shows the 20 most DEGs. Figure 5A shows the DEGs highlighted in red (higher in bronchial brushes) and blue (lower in bronchial brushes) in a scatter plot showing the mean expression in the bronchial and nasal brushes. Figure 5B shows a heatmap of the DEGs.

**Table 5:** Top 20 genes differentially expressed between nasal and bronchial brushes (|Log_2_FC| >1.5, FDR < 0.05).

Gene symbol	Log_2_FC	p-value	FDR
PAX6	3.270	3.09·10^−122^	3.05·10^−118^
CPA4	5.546	1.66·10^−122^	3.05·10^−118^
MUC21	4.275	1.46·10^−118^	9.62·10^−115^
GDPD3	3.689	7.20·10^−113^	3.55·10^−109^
CERS3	3.874	3.17·10^−109^	1.25·10^−105^
XDH	2.695	5.56·10^−109^	1.83·10^−105^
PAX3	2.726	2.69·10^−108^	7.59·10^−105^
VGLL1	3.188	1.98·10^−107^	4.88·10^−104^
C8orf34	−3.702	3.55·10^−106^	7.77·10^−103^
S100A4	2.902	8.67·10^−105^	1.71·10^−101^
PI3	3.726	2.65·10^−104^	4.76·10^−101^
SIX3	2.519	1.80·10^−103^	2.96·10^−100^
SPRR2A	6.237	2.15·10^−103^	3.26·10^−100^
FGF14	−4.115	2.88·10^−102^	4.05·10^−99^
CNTN3	−3.347	1.38·10^−101^	1.81·10^−98^
PAX7	2.797	8.88·10^−101^	1.09·10^−97^
GALNT14	2.166	1.95·10^−100^	2.26·10^−97^
ANKRD22	2.855	3.42·10^−100^	3.75·10^−97^
CD36	4.086	7.30·10^−100^	7.58·10^−97^
UCA1	5.230	9.80·10^−100^	9.66·10^−97^

### Network analysis on DEGs

From the 1692 DEGs, we built two GGM networks (one for each tissue) at BH-adjusted *p* ≤ 0.01. We found that 821 genes (48.52%) are connected in the bronchial network with 2642 edges (i.e. significant partial correlations), and 946 (55.91%) in nasal network with 5291 edges. We found that 1136 genes (67.13%) have at least one significant edge in one of the tissues, from which 179 genes (15.76%) show common edges, and the remaining 957 genes (84.24%) do not exhibit common edges.

**Figure 6:**
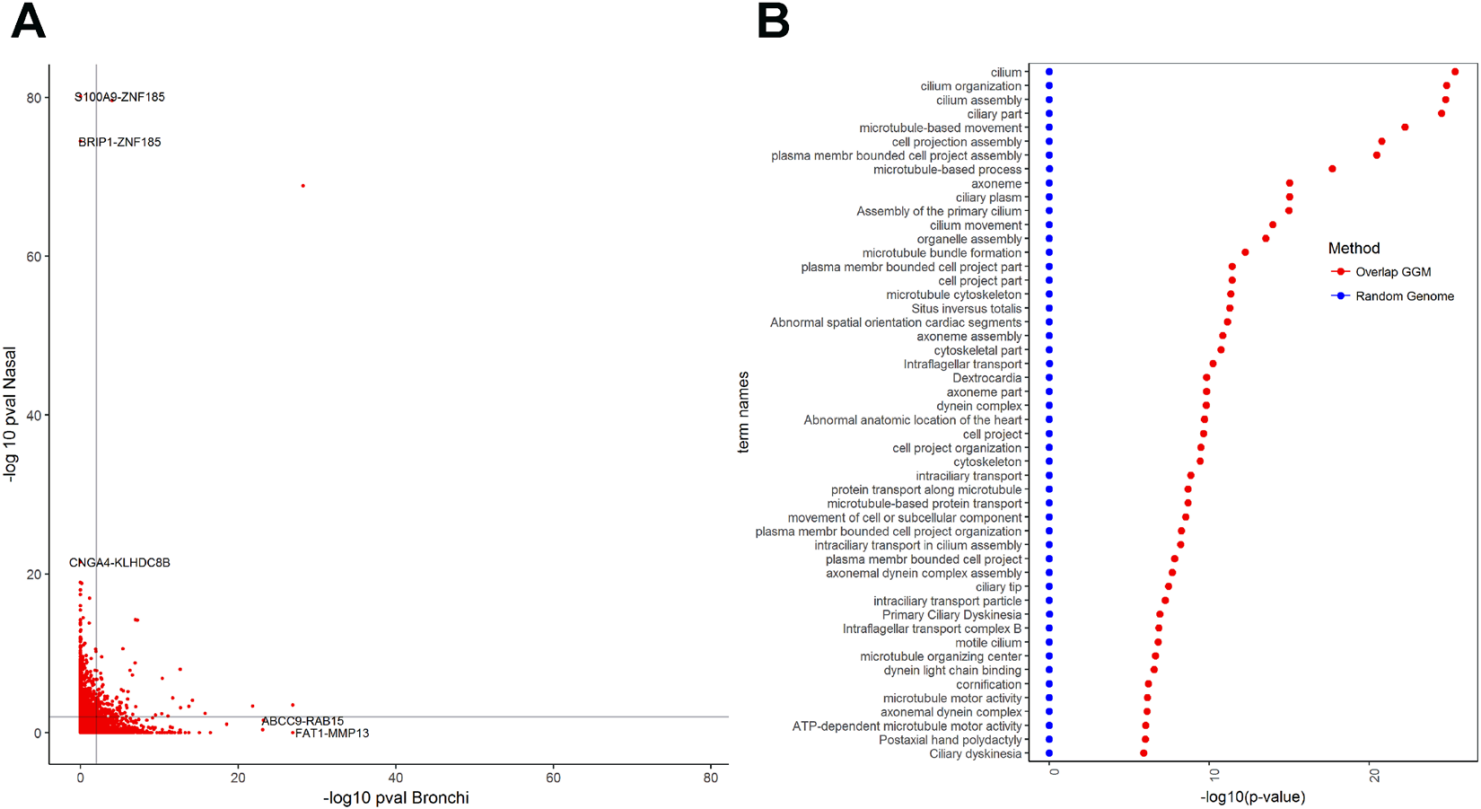
Network analyses for the differentially expressed genes (DEGs). (**A**) Scatter plot of the GGM network edges (partial correlation) for the DE genes. Each red dot represents $-lo{g}_{10}(\text{BH}\,p)$ of an edge in bronchial tissue (horizontal axis) versus nasal tissue (vertical axis). In light black: the critical value at BH $p\le \mathrm{0.01.}$ The figure displays the respective gene pairs for the most similar edges. (**B**) The 50 most significant GOs for the set of *Overlapped* genes. The panel displays the corresponding mean $-lo{g}_{10}$ (p-values), and error bars represent +2 standard errors over the 500 random samples.

Figure 6A shows an (edge-wise) comparison of the networks for each tissue via a scatter plot of BH p-values for the DEGs. This set of 957 genes will be referred to as the genes with non-overlapped edges. The gene ontology (GO) enrichment analysis identified 160 biological processes mainly related to cilium (e.g. cilium part, organization and assembly). The 50 most significant GOs are displayed in Fig. 6B. Table 6 shows the GO enrichment for biological processes at ${\rm{FDR}}\le 0.05$ for the non-overlapped genes.

**Table 6:** List of top 10 enriched GOs for the non-overlapping genes from the DEGs ($FDR\le 0.05$).

Term ID	Term name	p-value	Intersection
GO:0005929	cilium	4.27·10^−26^	PRKAR2B, DNAH9, MKS1, IFT88, DNAH5, CC2D2A, SPA17, IFT80, SPAG6, MOK, HSPB11, TEKT2, EFHC1, IFT27, HIF1A, GUCY2F, RPGRIP1L, CCDC114, DNAH11, TTC26, CEP41, B9D1, AKAP3, RSPH4A, MAK, NME5, IQCG, DNAH1, DNAH6, ALMS1, IFT46, DNAH7, IFT43, DNAL1, BBS9, IFT81, TTC21B, TEKT3, TAS2R4, TTBK2, TTLL9, TUBG1, MAP1B, CNGA4, DZIP1, TSGA10, TTLL7, DYNC2LI1, CCDC40, SPAG16, CCDC39, CETN2, WDR78, FAM49B, SPAG17, C8ORF37, WDR19, WDR66, RSPH1, SHANK2, BBS5, DNALI1, TMEM67, KIF27, AGBL2, TUB, DYNLRB2, TCTN2, ARL13B, TTLL6, DNAI2, CEP19, DNAH12, UNC119B, DNHD1, GPR157, MAATS1, AGBL4, DYNC2H1, IFT140, CERKL, SNTN, KIF19, TTC30B, DNAH10, CEP290, EFCAB7, TRAF3IP1, TCTEX1D2
GO:0044782	cilium organization	1.44·10^−25^	ZMYND10, PRKAR2B, FUZ, MKS1, IFT88, DNAH5, CC2D2A, IFT80, RFX3, PIH1D3, HSPB11, RFX2, TEKT2, IFT27, RPGRIP1L, CCDC114, TTC26, CEP41, B9D1, C11ORF63, RSPH4A, MAK, NME5, IQCG, DNAH1, DNAH6, ALMS1, IFT46, DNAH7, CNTRL, IFT43, DNAL1, BBS9, IFT81, TTC21B, TEKT3, TTBK2, FOXJ1, TUBG1, DZIP1, NEK1, DYNC2LI1, CCDC40, SPAG16, CCDC39, CETN2, SPAG17, WDR19, RSPH1, BBS5, STK36, TMEM67, KIF27, DNAAF2, PLK1, DNAAF3, DYNLRB2, TCTN2, ARL13B, DNAI2, UNC119B, DNHD1, CEP97, DYNC2H1, IFT140, KIF19, TTC30B, CEP290, TRAF3IP1, FGFR1OP, TCTEX1D2
GO:0060271	cilium assembly	1.69·10^−25^	ZMYND10, PRKAR2B, FUZ, MKS1, IFT88, DNAH5, CC2D2A, IFT80, RFX3, PIH1D3, HSPB11, RFX2, TEKT2, IFT27, RPGRIP1L, CCDC114, TTC26, CEP41, B9D1, C11ORF63, RSPH4A, MAK, NME5, IQCG, DNAH1, DNAH6, ALMS1, IFT46, DNAH7, CNTRL, IFT43, DNAL1, BBS9, IFT81, TTC21B, TEKT3, TTBK2, FOXJ1, TUBG1, DZIP1, NEK1, DYNC2LI1, CCDC40, SPAG16, CCDC39, CETN2, SPAG17, WDR19, RSPH1, BBS5, STK36, TMEM67, KIF27, DNAAF2, PLK1, DNAAF3, DYNLRB2, TCTN2, ARL13B, DNAI2, UNC119B, DNHD1, CEP97, DYNC2H1, IFT140, TTC30B, CEP290, TRAF3IP1, FGFR1OP, TCTEX1D2
GO:0044441	ciliary part	2.99·10^−25^	PRKAR2B, DNAH9, MKS1, IFT88, DNAH5, CC2D2A, SPA17, IFT80, SPAG6, MOK, HSPB11, EFHC1, IFT27, GUCY2F, RPGRIP1L, CCDC114, DNAH11, TTC26, CEP41, B9D1, AKAP3, RSPH4A, MAK, DNAH1, DNAH6, ALMS1, IFT46, DNAH7, IFT43, DNAL1, BBS9, IFT81, TTC21B, TAS2R4, TTBK2, TUBG1, MAP1B, CNGA4, DZIP1, DYNC2LI1, CCDC40, SPAG16, CCDC39, CETN2, WDR78, SPAG17, C8ORF37, WDR19, WDR66, RSPH1, SHANK2, BBS5, DNALI1, TMEM67, AGBL2, DYNLRB2, TCTN2, ARL13B, TTLL6, DNAI2, CEP19, UNC119B, DNHD1, GPR157, MAATS1, AGBL4, DYNC2H1, IFT140, CERKL, KIF19, TTC30B, DNAH10, CEP290, EFCAB7, TRAF3IP1, TCTEX1D2
GO:0007018	microtubule-based movement	5.74·10^−23^	DNAH9, IFT88, DNAH5, SPA17, IFT80, RFX3, PIH1D3, HSPB11, KIF9, TEKT2, IFT27, HIF1A, CCDC114, DNAH11, TTC26, RSPH4A, MAK, NME5, KIF20A, DNAH1, DNAH6, IFT46, DNAH7, IFT43, IFT81, TTC21B, TEKT3, DLGAP5, MAP1B, DYNC2LI1, CCDC40, SPAG16, CCDC39, WDR78, UCHL1, SPAG17, FMN2, WDR19, AP3S2, WDR66, WDR63, STK36, KIF6, KIF27, DPCD, DYNLRB2, TTLL6, DNAI2, DNAH12, DNHD1, MAATS1, DYNC2H1, IFT140, KIF19, TTC30B, DNAH10, TRAF3IP1, TCTEX1D2
GO:0030031	cell projection assembly	1.63·10^−21^	ZMYND10, PRKAR2B, FUZ, MKS1, IFT88, VCL, DNAH5, CC2D2A, IFT80, FSCN1, RFX3, PIH1D3, HSPB11, RFX2, TEKT2, IFT27, RPGRIP1L, CCDC114, TTC26, CEP41, B9D1, PMP22, C11ORF63, RSPH4A, MAK, NME5, DPYSL3, IQCG, ARHGEF26, DNAH1, DNAH6, ALMS1, IFT46, DNAH7, CNTRL, IFT43, DNAL1, BBS9, IFT81, TTC21B, TEKT3, CDC42EP1, TTBK2, FOXJ1, TUBG1, EMP1, DZIP1, TSGA10, NEK1, DYNC2LI1, CCDC40, SPAG16, CCDC39, CETN2, SPAG17, KIT, WDR19, RSPH1, BBS5, STK36, TMEM67, KIF27, DNAAF2, PLK1, DNAAF3, DYNLRB2, TCTN2, ARL13B, LPAR3, DNAI2, UNC119B, DNHD1, CEP97, DYNC2H1, IFT140, TTC30B, CEP290, TRAF3IP1, FGFR1OP, TCTEX1D2, KLHL41
GO:0120031	plasma membrane bounded cell projection assembly	3.46·10^−21^	ZMYND10, PRKAR2B, FUZ, MKS1, IFT88, VCL, DNAH5, CC2D2A, IFT80, FSCN1, RFX3, PIH1D3, HSPB11, RFX2, TEKT2, IFT27, RPGRIP1L, CCDC114, TTC26, CEP41, B9D1, PMP22, C11ORF63, RSPH4A, MAK, NME5, DPYSL3, IQCG, ARHGEF26, DNAH1, DNAH6, ALMS1, IFT46, DNAH7, CNTRL, IFT43, DNAL1, BBS9, IFT81, TTC21B, TEKT3, CDC42EP1, TTBK2, FOXJ1, TUBG1, EMP1, DZIP1, NEK1, DYNC2LI1, CCDC40, SPAG16, CCDC39, CETN2, SPAG17, KIT, WDR19, RSPH1, BBS5, STK36, TMEM67, KIF27, DNAAF2, PLK1, DNAAF3, DYNLRB2, TCTN2, ARL13B, LPAR3, DNAI2, UNC119B, DNHD1, CEP97, DYNC2H1, IFT140, TTC30B, CEP290, TRAF3IP1, FGFR1OP, TCTEX1D2, KLHL41
GO:0007017	microtubule-based process	2.01·10^−18^	ZMYND10, DNAH9, IFT88, DNAH5, CC2D2A, SPA17, ASPM, IFT80, RFX3, PIH1D3, HSPB11, KIF9, TEKT2, IFT27, HIF1A, KATNAL1, CCDC114, DNAH11, TTC26, C11ORF63, RSPH4A, MAK, NME5, KIF20A, IQCG, DNAH1, DNAH6, CDC20, IFT46, DNAH7, IFT43, DNAL1, IFT81, TTC21B, TEKT3, DLGAP5, GNAI1, TUBG1, MAP1B, GCC2, DYNC2LI1, CCDC40, SPAG16, CCDC39, PLK2, CETN2, CHEK1, DIXDC1, WDR78, UCHL1, SPAG17, FMN2, WDR19, AP3S2, WDR66, TPPP3, RSPH1, CCDC78, WDR63, STK36, SPICE1, KIF6, TMEM67, KIF27, MELK, DNAAF2, DPCD, PLK1, MAP1A, DNAAF3, DYNLRB2, TTLL6, MAP6, DNAI2, CNP, DNAH12, DNHD1, CEP97, MAATS1, DYNC2H1, IFT140, KIF19, TTC30B, DNAH10, TRAF3IP1, FGFR1OP, TCTEX1D2
GO:0097014	ciliary plasm	9.55·10^−16^	DNAH9, DNAH5, SPAG6, EFHC1, RPGRIP1L, CCDC114, DNAH11, RSPH4A, DNAH1, DNAH6, DNAH7, DNAL1, DYNC2LI1, CCDC40, SPAG16, CCDC39, WDR78, SPAG17, WDR66, BBS5, DNALI1, DYNLRB2, ARL13B, DNAI2, DNHD1, MAATS1, DYNC2H1, IFT140, KIF19, DNAH10, TRAF3IP1, TCTEX1D2
GO:0005930	axoneme	9.55^−16^	DNAH9, DNAH5, SPAG6, EFHC1, RPGRIP1L, CCDC114, DNAH11, RSPH4A, DNAH1, DNAH6, DNAH7, DNAL1, DYNC2LI1, CCDC40, SPAG16, CCDC39, WDR78, SPAG17, WDR66, BBS5, DNALI1, DYNLRB2, ARL13B, DNAI2, DNHD1, MAATS1, DYNC2H1, IFT140, KIF19, DNAH10, TRAF3IP1, TCTEX1D2

### Cell type decomposition and gene set variation analysis (GSVA) analysis

**Figure 7:**
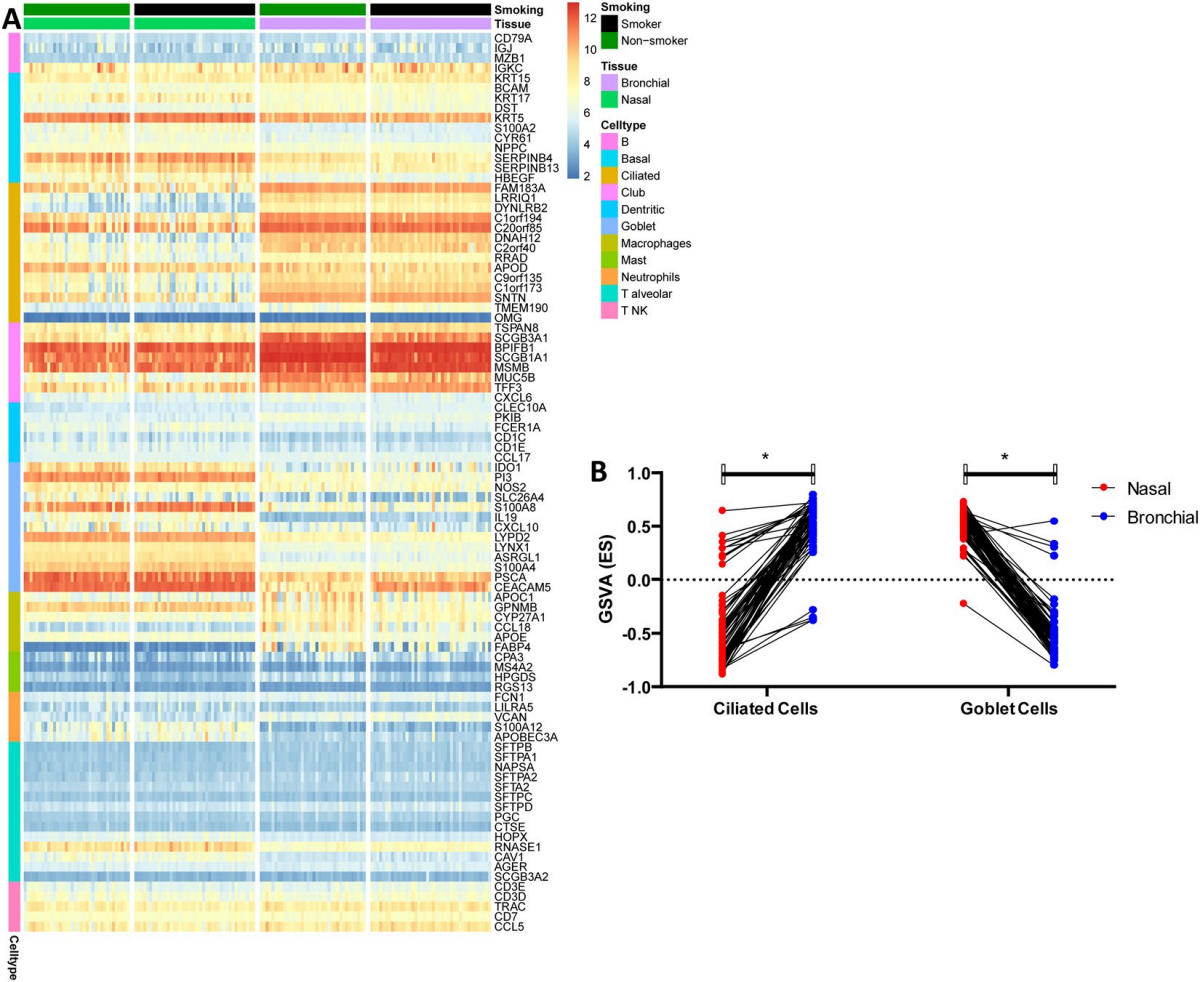
Cell type-specific marker analysis between bronchial and nasal epithelium. In panels of this figure, we investigate the difference in cell-type composition. Panel (A) shows the expression profile of genes identified in recent single-cell profiling of human lungs^23^ in a form of heatmap stratified by tissue type and smoking status. Bronchial epithelium shows higher expression of genes that mark Ciliated and Club cells, while nasal tissue exhibits higher expression of genes characteristic for Goblet cells. Within tissue expression patterns of marker genes is similar between smoker and never smokers where apparent difference can be observed only at the level of individual genes (e.g. MUC5B or CEACAM5). Panel (B) GSVA of ciliated and goblet cell genes from match nasal and bronchial brushes. The scatter plot shows the overall lower GSVA scores of different gene sets attributed to Ciliated cells in nasal samples and the overall higher GSVA scores of gene sets corresponding to Goblet cells.

To investigate the difference in cell-type composition between bronchial and nasal epithelium, we employed markers identified in recent single-cell profiling of human lungs^23^. Interestingly, bronchial epithelium shows higher expression of genes that mark Ciliated and Club cells, while nasal tissue exhibits higher expression of genes characteristic for Goblet cells (Fig. 7A). Within tissue the expression patterns of marker genes is similar between smoker and never smokers where apparent difference can be observed only at the level of individual genes (e.g. MUC5B or CEACAM5). In addition to determine differential pathways expression we have performed GSVA of both tissue type using gene sets attributed to Goblet, and Ciliated cells (Fig. 7B).

## Discussion

In the current study, we used a network-based approach to identify pathways that explain the differences and similarities between the gene expression profiles from nasal and bronchial brushes. This is the first study using GGMs to compare gene networks from the expression data obtained from nasal and bronchial samples. We show that genes involved in inflammatory pathways are retained between the two compartments, while cilia-related genes are lower expressed in the nose. The detoxifying gene expression response of the respiratory tract to cigarette smoking is similar in the nose and bronchus.

When investigating pathways from genes with similar expression in the nose and bronchus, we found genes involved in pro-inflammatory pathways, including the HLA-family, *CFD*, *CD207* and *MT2A*. The HLA class II molecules have a key function in the adaptive immune response by providing peptides to the antigen receptor of CD4 +T lymphocytes. Several studies have found an association between Human Leukocyte Antigen (HLA) class II genes and asthma, as well as with other allergic diseases, such as allergic rhinitis and atopic dermatitis^22^. Previous studies have shown similar eosinophilic infiltration (a hallmark of inflammation in asthma) in the nose and bronchus of healthy controls and asthmatics on corticosteroid therapy. Interestingly, although increased inflammation in untreated asthmatics compared to healthy controls was present in both compartments, the inflammation was further enhanced in the bronchus. Furthermore, *in vitro* studies have shown high concordance in inflammatory responses to the pro-inflammatory stimuli *IL-1β*, *TNF-α* and rhinovirus in both nasal and bronchial epithelium^24,25^. The strong concordance in gene expression networks for genes involved in pro-inflammatory pathways (between samples collected in the nose and the bronchus) indicates that the nasal epithelium could be used to study underlying molecular mechanisms of inflammation and to identify easily accessible biomarkers for chronic inflammatory disease classification.

Furthermore, when looking at pathways concordantly affected by smoking between the nose and the bronchus, genes providing proteins with oxidoreductase activity such as the cytochrome P450 genes (e.g. *CYP1A1*, *CYP1B1*) and *ALDH1A3*, had similar expression between the nose and the bronchus. Previously, it has been shown that genes induced by smoking and having oxidoreductase activity are one of the most rapidly reversible genes upon smoking cessation^8^. Our findings further support the idea of a detoxifying response to tobacco exposure throughout the airways in smokers. Previous studies focusing on *ex vivo* nasal and bronchial biopsies have shown similar response to cigarette smoke stimulation between the two tissues^26^. Moreover, current and former smokers’ bronchial epithelial gene expression has been used to derive and validate a biomarker to detect lung cancer^27^. Subsequently, it was found that lung cancer-associated gene expression was detectable from nasal epithelium, with both tissue showing concordant expression alterations (e.g. regulation of apoptosis and immune system signaling)^28^.

Another gene identified in our analysis is *TFF1*, which encodes for the trefoil factor family peptides. These peptides are secreted by mucus-producing cells into mucosal surfaces throughout the body and can bind to mucin molecules. It has been shown in mouse studies that *TFF1* expression is increased after injury of the airway epithelium, suggesting a role in airway epithelial repair^29^. Another interesting gene is *CTNNAL1*, a gene that has been associated with cellular growth regulation and may be involved in the recovery of (bronchial) epithelial damage^30^.

A number of genes were found to have different expression levels at baseline in nasal and bronchial airway epithelium. However, we still found an overlap between the two networks, indicating that regardless of the different baseline levels of individual genes, there may still be correlations for the gene-sets as a whole between the two compartments. Similar findings have been observed in *in vitro* models, showing different levels of certain genes between nasal and bronchial epithelial cultures, and nevertheless, these genes still correlated in their response to stimuli^25^. Previous studies focusing on ciliary beat frequency found that this was similar in nasal and bronchial epithelium^31^. However, pathway analysis of differentially expressed genes between nose and bronchus in our study indicated cilia-related pathways to be decreased in nasal compared to bronchial brushes. In line with this observation, it has been shown that the percentage of ciliated cells increases further down the respiratory tract as shown by a previous study^32^. This change in percentage will lead to a shift in cellular composition and an increase of cilia-related gene expression in the lower airways. This finding was confirmed by our GSVA were nasal epithelium showed lower expression for ciliated genes compared to bronchial epithelium. These results support also the high quality of our nasal and bronchial epithelium dataset.

The strength of our study is the use of matched nasal and bronchial samples from a moderately powered dataset. There are also a number of limitations associated with this study. First, from the network analysis perspective GGMs are limited to linear associations (partial correlation). Therefore, if a pair of genes shows a nonlinear relationship, the GGM might not identify these associations. Second, network inference is a multiple testing scenario, i.e. p genes imply performing *p(p-1)*/*2* tests. Here, we have employed BH correction for multiple testing, however, it should be kept in mind that false positives (false edges) might be still present in the network structure regardless of the method depending on the chosen FDR error tolerance. Third, the small number of genes used for the enrichment analysis may have influenced the pathways we found. A subset of the genes with similar gene expression patterns between nasal and bronchial brushing identified in our current analysis are likely due to genetic polymorphisms that have effects on gene expression in general through the whole body not exclusively related to the tissues measured in the current manuscript.

In conclusion, we have shown that although there are differences in expression between the nasal and bronchial brushes, their response to external factors such as smoking seem to be concordant. Therefore, we suggest that the use of nasal brushes as a proxy for the bronchus is suitable to study airway epithelium at baseline and in response to environmental exposures.

## Materials and Methods

### Study participants and data processing

Data were collected from participants from the study to obtain NORMal values of inflammatory variables from healthy subjects (NORM) NCT00848406 and included healthy smokers and never-smokers. Details on the methods and study design have been previously published^22^. Bronchial and nasal brushings were collected during the same visit, using a Cellebrity bronchial brush (Boston Scientific, Massachusetts, USA) or a Cyto-Pak CytoSoft nasal brush (Medical Packaging Corporation, Camarillo, CA, USA). Samples were then randomized, labeled and run on Affymetrix Human Gene chip ST1.0 arrays as described previously according to manufacturer’s instructions (Thermo Fisher Scientific, Waltham, Massachusetts, USA)^22^. Microarray analyses were performed using R (v3.3.2) limma package and normalization was conducted in a single batch using Robust Multi-array Average (RMA). The study procedures were approved by the local medical ethics committee (Medisch Ethische Toetsingsingscommissie or METc) and written informed consent was given by all subjects. All experiments in this study were performed in accordance with relevant Dutch national and international guidelines and regulations.

### Gene expression analysis

For the nasal bronchial comparison, a gene was determined to be expressed if the median log_2_(normalized microarray florescence intensity) >3.

To identify concordant genes where variation among subjects was correlated between sampling locations, we applied a Spearman correlation comparing the expression of individual genes between nasal and bronchial brushes. To assess whether the nasal-bronchial relation within a patient was stronger than across patients, we applied a Spearman correlation on all paired samples and compared this to the average correlation on mismatched pairs. This analysis was conducted on all genes and on the correlated genes.

In order to identify genes differentially expressed between nasal and bronchial brushes, we performed a paired analysis, using a linear model correcting for age, gender and smoking status. Genes were considered significantly differentially expressed if their BH corrected p-value was <0.05 and if they had a |log fold change| > 2.

### Network analysis

Three different sets of genes were studied: *(i)* the set of CO, *(ii)* twenty seven smoking related genes previously found to be differentially expressed in both the nose and the bronchus^22^ (SM), and *(iii)* the set of DEGs. The expression data has been log_2_-tranformed and standardized (mean = 0, sd = 1) such that the data is normal distributed.

As our dataset involves a number of genes that is larger than the sample size, the reconstruction of a GGM (i.e. inferring the partial correlations) from expression data requires a shrinkage approach. This is usually known as a high-dimensional problem. To this end, the network analysis was performed using the R package *GeneNet* version 1.2.13^12^ in R version 3.4.3. The test of significance for each of the GGM’s edges (i.e. the partial correlations) was performed with the improved method that we have published recently in *Oxford* Bioinformatics^33^. This method test the null-hypothesis of no partial correlation between pairs of genes, and has an accurate control of the false positives. For networks involving *p* number of genes there are *p(p-1)/2* edges to test which is a multiple testing problem, thus the significance of the edges was adjusted with BH approach.

### Gene ontology enrichment analysis

GO enrichment analysis was performed with the R package *gProfileR* version 0.6.4^34^, and its default “ontology-focused” multiple testing correction (i.e. **g:SCS**)^35^. The resulting GO enrichment was assessed by employing a gene sampling procedure. First, we identify which of the GGM’s edges were common in both of the analyzed tissues and the corresponding genes were checked for GO enrichment. These pairs of genes (i.e. from the common edges in nasal and bronchial networks) are denoted as the overlapped genes. Second, the GO enrichment from the overlapped genes are compared against the GO enrichment from two others sets of genes (of same size) obtained randomly. The genes of these random sets are sampled from *(i)* the whole set of protein-coding genes (19718 genes), and from *(ii)* their corresponding gene sets (e.g. CO, SM or DEG). If the number of connected genes in the network was greater than 50% its corresponding set (e.g. 50% of the genes in CO, SM or DEG) the sampling was omitted as the two sets are considered similar.
